# Early multimodal rehabilitation and functional outcomes of a left brachial plexus injury after general anesthesia: a case report

**DOI:** 10.3389/fresc.2026.1765023

**Published:** 2026-06-03

**Authors:** Yiru Jiang, Shuqi Hu, Bingjie Yang, Wei Zhang, Song Shu, Hao Zhang

**Affiliations:** 1 The Fourth School of Clinical Medicine, Zhejiang Chinese Medical University, Hangzhou First People's Hospital, Hangzhou, China; 2 Department of Neurology, Affiliated Hangzhou First People's Hospital, School of Medicine, Westlake University, Hangzhou, China; 3 Motor Neuron Disease Diagnosis and Treatment Center, Affiliated Hangzhou First People's Hospital, School of Medicine, Westlake University, Hangzhou, China

**Keywords:** brachial plexus injury, functional rehabilitation, general anesthesia, peripheral nerve injury, peripheral nerve regeneration

## Abstract

Brachial plexus injury (BPI) is a common perioperative complication, often caused by intraoperative trauma or improper positioning during surgery. While some BPIs recover spontaneously, many patients experience long-term functional impairments, particularly in the upper limb. This case is distinguished by its focus on a rare perioperative iatrogenic C5–C6 BPI in an adolescent following laparoscopic surgery. Crucially, unlike many traditional protocols, an early multimodal rehabilitation program was implemented within only one week of diagnosis. This program incorporated physical therapy, neuromuscular electrical stimulation, and progressive resistance training. After six months, the patient achieved full motor recovery and regained unrestricted mobility in his left upper limb. This case highlights the importance of very early intervention in optimizing functional outcomes and effectively preventing secondary complications like muscle atrophy, even in patients with potential for spontaneous recovery.

## 1 Introduction

Perioperative peripheral nerve injury (PNI), a recognized complication of general anesthesia, presents symptoms and signs including numbness, paresthesia, tingling, pain, or muscle weakness. It may result from patient comorbidities, intraoperative positioning, and surgical factors (1–3).

Iatrogenic brachial plexus injury (BPI), a common perioperative PNI, stems from the brachial plexus's susceptibility to intraoperative positioning, stretch, and compression (4). The American Society of Anesthesiologists Closed Claims Project noted that 15% of claims involved anesthesia-related nerve injuries, of which BPIs accounted for 23% (1). While preventive measures can reduce BPI incidence, complete prevention remains unachievable. Crucially, BPIs often cause severe upper limb sensorimotor disturbances and muscle loss, with a 75% disability rate—significantly higher than the 60% rate for general PNIs (3, 5)—making prompt, targeted early rehabilitation critical for mitigating sequelae like muscle atrophy (3).

A key clinical challenge is the lack of standardized comprehensive rehabilitation protocols for perioperative BPI, hindered by limited high-quality research (6). While Smania et al. emphasize that multimodal approaches—integrating non-pharmacological methods like transcutaneous electrical nerve stimulation (TENS) with targeted physical therapy—are crucial, such strategies remain significantly underexplored in clinical practice (3).

Currently, reports specifically addressing early multimodal rehabilitation for BPI following general anesthesia are exceedingly scarce, restricting clinical guidance for optimizing patient recovery. This case details the complete restoration of upper limb function in a patient with BPI post-general anesthesia through early multimodal rehabilitation. Therefore, the purpose of this case report is to describe a comprehensive, evidence-based multimodal rehabilitation program, including the clinical examination, intervention rationale, and functional outcomes for a patient with iatrogenic BPI.

## 2 Case presentation

### 2.1 Patient information

A 15-year-old right-handed male underwent a 4-hour laparoscopic appendectomy under general anaesthesia while maintained in a supine position with modified Trendelenburg positioning. Upon awakening, he immediately experienced an inability to lift his left upper limb. The patient recalled that he retained normal, pain-free movement in his fingers and left wrist following surgery. The patient had no prior history of similar symptoms, recent trauma, or peripheral nerve disorders. There was no relevant genetic or psychosocial history. Additionally, the patient had no other underlying medical conditions, and no history of long-term medication use, exposure to toxic or hazardous substances, or smoking or alcohol consumption. The symptoms were initially regarded as a typical postoperative occurrence following general anesthesia, anticipated to resolve spontaneously. Consequently, the patient did not receive prompt post-procedural treatment.

**Figure 1:**
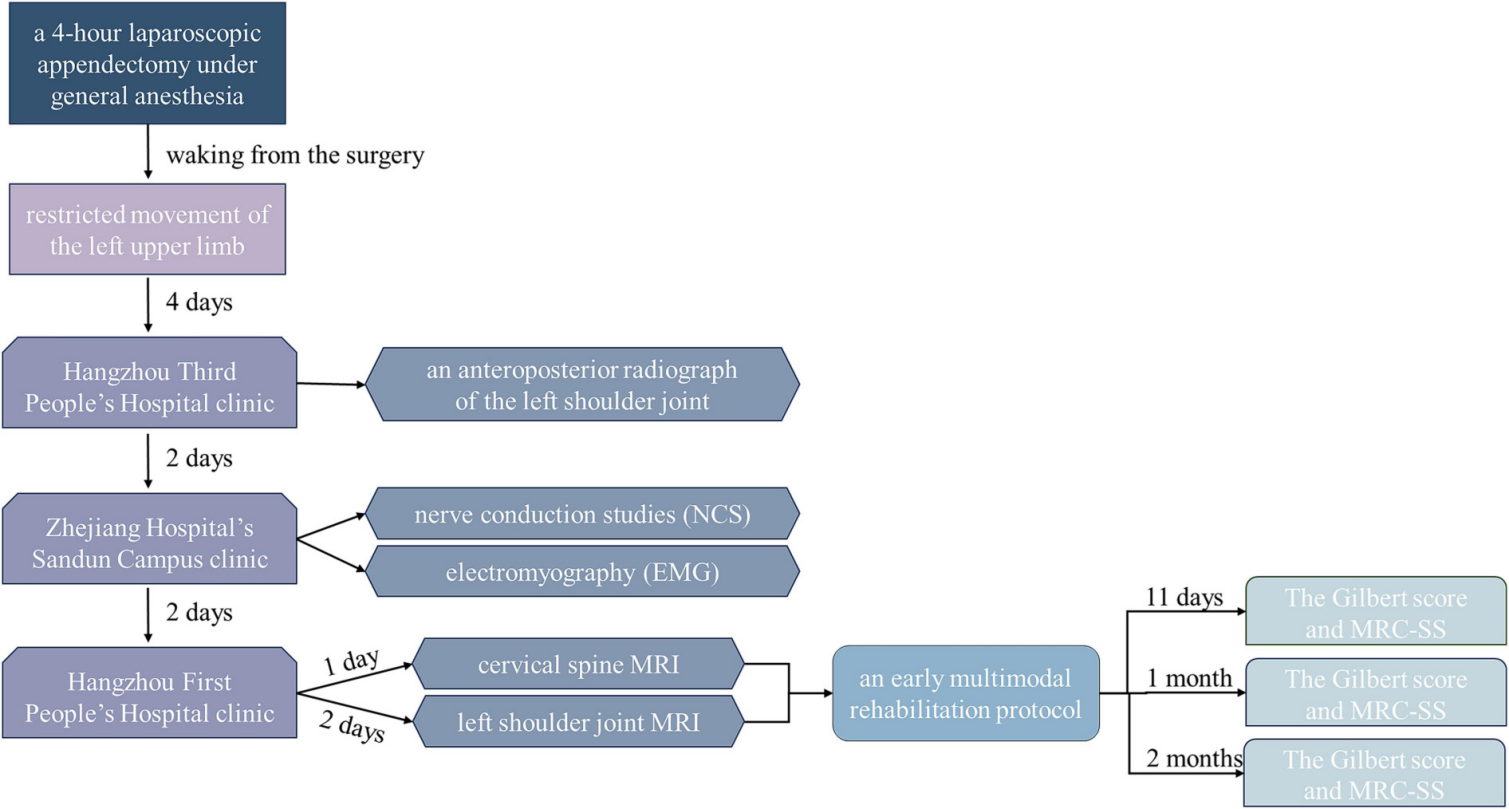
Timeline of the patient's episode of care. The timeline details key events from the initial surgery to diagnostic procedures (radiograph, NCS/EMG, MRI) and the schedule of rehabilitation assessments (Gilbert score and MRC-SS) over a two-month period. MRC-SS, medical research council sum-score; MRI magnetic resonance imaging.

Approximately a week post-surgery, the patient's left upper limb weakness persisted, impairing daily activities and school participation. This ongoing issue raised concerns for the patient and his parents regarding potential effects on long-term physical growth and development, leading them to pursue further evaluation and treatment at multiple medical facilities. Despite adhering to medical advice and using neurotrophic medications, the patient experienced unsatisfactory outcomes. Subsequently, the patient pursued additional treatment at our hospital's neurology outpatient clinic. Figure 1 illustrates the timeline of events for this patient.

### 2.2 Clinical and physiotherapy findings

During the initial visit to our outpatient clinic, the patient was unable to elevate the left upper limb or flex the left elbow joint. A comprehensive neurological examination was subsequently performed.

Physical examination of the left upper limb revealed decreased muscle tone, with preserved metacarpophalangeal extension, wrist flexion and extension, grip strength, and sensation.

Manual muscle testing demonstrated marked weakness, with the following grades (out of 5): shoulder flexion (1/5), shoulder abduction (0/5), shoulder extension (2/5), shoulder elevation (0/5), elbow flexion (0/5), and elbow extension (3/5). Biceps, triceps, and brachioradialis reflexes were diminished (+), and left-sided Hoffmann and Babinski signs were negative. Coordination and balance were intact, cardiopulmonary auscultation was unremarkable, the abdomen was soft and nontender without rebound pain, and no lower extremity edema was observed. The comprehensive clinical findings from the physical examination are summarized in Table 1.

**Table 1:** Clinical findings that emerged during the physical examination.

Category	Assessment	Patient Findings
General condition	Body weight	83 kg
	Height	173 cm
Vital signs	Temperature	36.6℃
	Blood pressure	150/93mmHg
	Heart rate values	110 beats per minute.
Neurological examination	Muscle tone	Reduced
	Metacarpophalangeal extension	WNL
	Wrist flexion	WNL
	Wrist extension	WNL
	Grip strength	WNL
	Upper limb sensation	WNL
	Shoulder flexion	1/5
	Shoulder abduction	0/5
	Shoulder extension	2/5
	Shoulder elevation	0/5
	Elbow flexion	0/5
	Elbow extension	3/5
	Biceps reflex	↓
	Triceps reflex	↓
	Radial periosteal reflex	↓
	Hoffmann sign (left)	Negative
	Babinski sign (left)	Negative
	Coordination	Intact
	Balance	Intact
Other physical examination	Cardiopulmonary auscultation	Unremarkable
	Abdomen	Soft, nontender, no rebound pain
	Lower extremity edema	Absent

WNL, within normal limits; ↓ diminished.

The C5–C6 nerve roots primarily innervate the shoulder and proximal upper limb muscles (7). The patient's inability to elevate the left upper limb, coupled with marked weakness in the shoulder and proximal upper limb muscles, diminished reflexes, and intact sensory function, strongly suggests a C5–C6 nerve root or upper trunk brachial plexus injury. The C7 nerve root, which contributes to the radial nerve, innervates the upper limb extensor muscles responsible for elbow, wrist, and finger extension (8). The preservation of wrist and finger movements indicates that the C7, C8, and T1 nerve roots are intact, or that possible C7 damage is being compensated by functional C6 and C8 nerve roots.

### 2.3 Diagnostic assessment

Before admission to our facility, the patient underwent several diagnostic evaluations at multiple hospitals. On the fourth postoperative day, an anteroposterior radiograph of the left shoulder joint at Hangzhou Third People's Hospital revealed no significant bony abnormalities. Although a definitive diagnosis of BPI was not initially established, these findings helped exclude bony etiologies and pointed toward a neurogenic injury.

Six days post-operation, nerve conduction studies (NCS) and needle electromyography (EMG) conducted at Zhejiang Hospital showed normal sensory conduction but reduced evoked potential amplitudes in the left radial nerve. Needle EMG demonstrated neurogenic damage in the biceps, deltoid, trapezius, and infraspinatus muscles.

**Figure 2:**
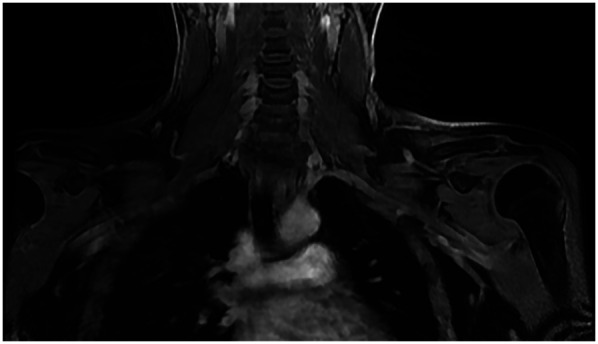
MRI of cervical spine (sagittal T2-weighted turbo spin-echo). The image shows reversed cervical lordosis (straightened curvature). Critically, no spinal cord compression, disc herniation, or nerve root avulsion is observed, which helped exclude structural cervical spine pathology as the cause of the patient's neurological deficits.

**Figure 3:**
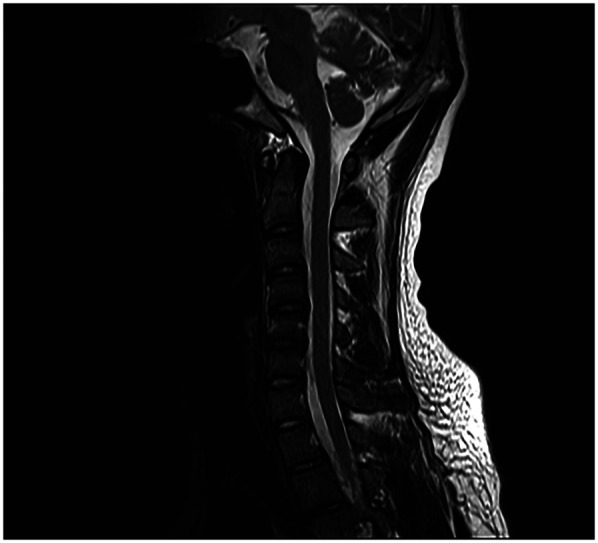
MRI of the left shoulder (WATER: BH Cor LAVA-flex + C). This MRI, performed 10 days post-surgery, shows no notable morphological abnormalities in the bilateral brachial plexus nerves. Both shoulder joints and their associated soft tissues also appeared morphologically normal, ruling out structural causes like masses or ruptures.

The patient was admitted to our institution on day eight presenting with restricted left upper limb movement. Given the clinical history, cerebrovascular disease was considered unlikely, and cranial magnetic resonance imaging (MRI) was deferred in favor of targeted cervical spine and left shoulder joint MRI. Cervical spine MRI (day 9) revealed a reversed cervical curvature but no injury-related abnormalities, effectively excluding spinal cord or nerve root lesions (Figure 2). Left shoulder joint MRI (day 10) showed no notable morphological abnormalities in the bilateral brachial plexus (Figure 3; Table 2). Furthermore, laboratory blood tests were unremarkable for metabolic, inflammatory, or infectious etiologies. Based on the integrated clinical and electrophysiological findings, the patient was diagnosed with a left upper trunk brachial plexus injury (C5–C6).

**Table 2:** Magnetic resonance imaging protocol.

Region	Plane	Sequence	Repetition time (TR, ms)	Echo time (TE, ms)	Slice thickness (mm)	Field of view (mm × mm)
cervical spine	Sagittal	T1-TSE sag 320	396	9.6	3.5	270 × 243
	Sagittal	T2-TSE RT sag 384	2,070	102	3.5	270 × 244.7
	Transverse	T2-TSE RT MSMA tra 384	2,930	111	3.5	180 × 163.1
	Sagittal	Localizer pat2	35	5.86	10	300 × 300
shoulder joint	Coronal	OCOr STIR	4,772	56.1	3	300 × 300
			5,122	57.408	4	300 × 300
			5,123	57.408	3	300 × 300
			5,131	57.564	3	300 × 300
			5,277	51.832	4	360 × 360
		WATER: BH Cor LAVA-Flex + C	6.384	3.126	3	400 × 400
	Axial	OAx T2 IDEAL shim	3,579	73.872	3	300 × 300

T1-TSE sag 320 T1-weighted Turbo Spin Echo sagittal 320 matrix; T2-TSE RT sag 384 T2-weighted turbo spin echo with respiratory-triggered sagittal 384 matrix; T2-TSE RT MSMA tra 384 T2-weighted turbo spin echo with respiratory-triggered multi-slab multi-angle transverse 384 matrix; Localizer pat2 Localizer acquisition (pat2); OCOr STIR oblique coronal short-tau inversion recovery; OAx T2 IDEAL shim oblique axial T2-weighted IDEAL shimming; WATER, BH Cor LAVA-Flex + C water-only image from breath-hold coronal LAVA-flex with contrast.

### 2.4 Treatment, intervention rationale, and outcomes

Prior to receiving treatment at our institution, the patient underwent pharmacological therapy at another medical facility. Six days post-operation, the patient consulted Zhejiang Hospital's Sandun Campus clinic, received methylcobalamin treatment (0.5 mg three times daily), but the symptoms showed no significant improvement. This caused considerable anxiety and distress for the patient and their family.

Upon admission, we promptly initiated neurotrophic therapy, such as Adenosine Cobalamin (1.5 mg daily), Mouse Nerve Growth Factor (30 μg daily) and Ganglioside (20 mg daily), while concurrently administering Dexamethasone (10 mg daily) for anti-inflammatory treatment over five days.

During hospitalization, the patient received regular supervised rehabilitation sessions, usually once daily on weekdays (approximately 5 days per week), with each session lasting about 30–40 min. The program included passive range-of-motion (PROM) and active-assisted range-of-motion (AAROM) exercises of the shoulder and elbow, performed within a pain-free range to maintain joint integrity, prevent soft tissue contractures, and provide sensory input in the early stage of nerve injury (6). TENS was applied to the deltoid and biceps muscles. In this case, stimulation was delivered at a frequency of 35 Hz with a pulse width of 200 μs for 20 min daily to facilitate muscle re-education and reduce disuse atrophy (3). Therapist-guided exercises were also provided to encourage active muscle recruitment and improve motor control.

In the early stage, rehabilitation was mainly therapist-guided, with a smaller component consisting of simplified home exercises. As motor function improved, home-based exercise gradually became a larger part of the program. Supervised rehabilitation was continued throughout the inpatient period (approximately 2 weeks), followed by a structured home exercise program with periodic follow-up.

The progression from assisted exercise to resistance training was based on recovery of active movement against gravity (MRC grade ≥3), improvement in pain-free joint range of motion, and the ability to perform repeated movements without excessive fatigue. Once these criteria were met, low-intensity resistance training with elastic bands was introduced, typically 3–5 times per week, with the volume adjusted according to the patient's recovery status and tolerance.

During rehabilitation, the patient exercised his left upper extremity and observed its movements under the guidance of a qualified therapist. This physiotherapy was performed intermittently in our therapy pool. Follow-up assessments using the Gilbert score and Medical Research Council Sum-Score (MRC-SS) were conducted to assess shoulder and elbow outcomes (Table 3). The Gilbert score is a standardized tool used specifically to evaluate shoulder function following brachial plexus injuries, with grades ranging from 0 (complete flail shoulder) to 5 (normal function with active abduction > 120°) (9). The MRC-SS assesses muscle strength on a 6-point scale, where 0 indicates no visible or palpable muscle contraction, and 5 represents normal strength against full resistance (10). Shoulder abduction range of motion was measured in degrees against gravity. The hand-on-abdomen position was considered −90 degrees when measuring shoulder external rotation range of motion with gravity eliminated.

**Table 3:** MRC-SS and gilbert scores in patients with BPI during rehabilitation.

Item	Region	Movement	Timepoint			
			baseline (T0)	11 days (T1)	1 month (T2)	2 months (T3)
MRC-SS	Shoulder	Flexion	1	2	3	5
		Abduction	0	2	3	5
		Extension	2	2	3	5
		Lift	0	0	0	5
	Elbow	Flexion	0	2	3	5
		Extension	3	4	5	5
Gilbert score	/	/	0	1	3	5

MRC-SS, medical research council sum-score; BPI, brachial plexus injury.

**Figure 4:**
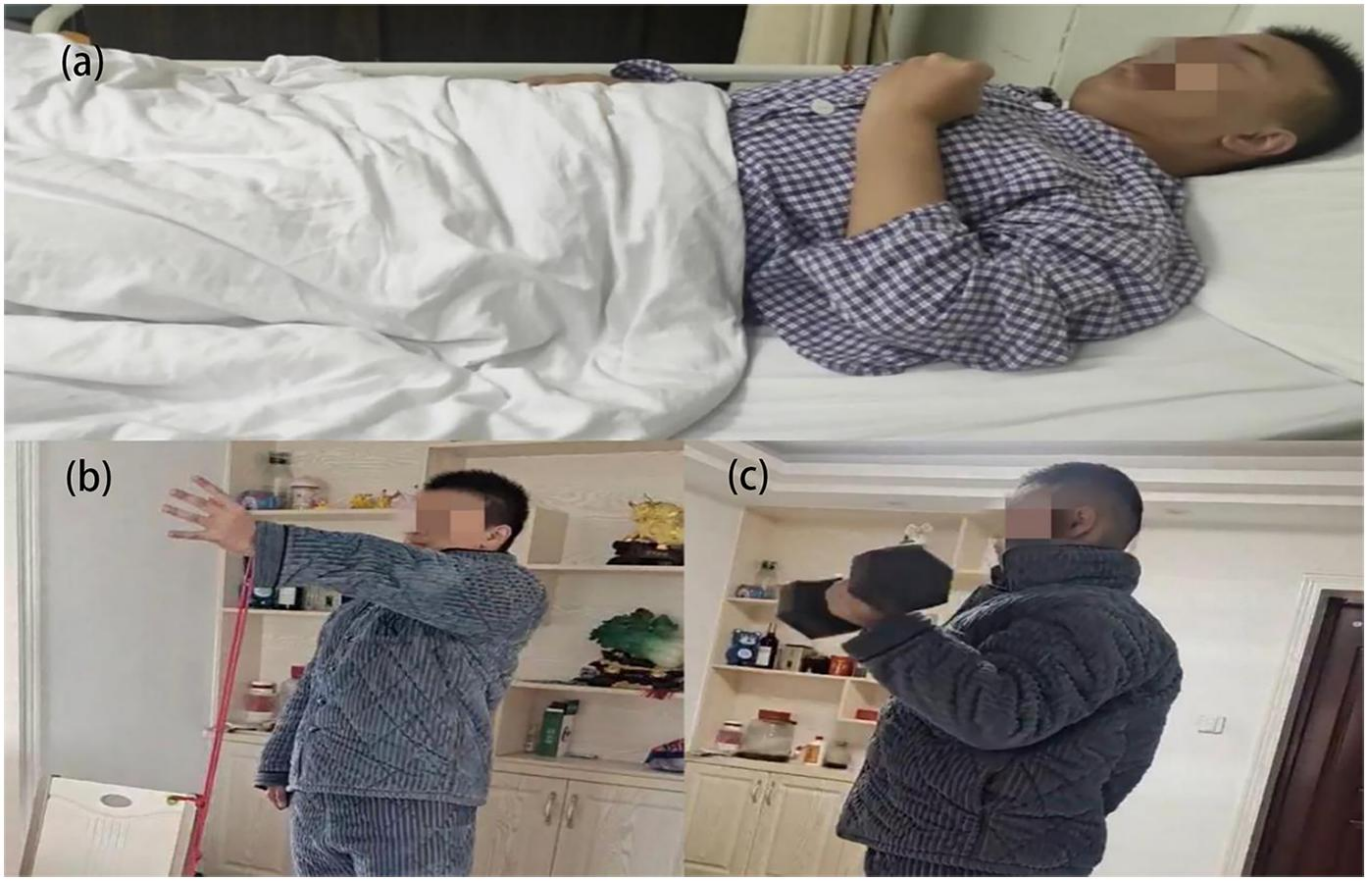
Photographic documentation of the patient's rehabilitation training process. **(a)** Inpatient session: patient performing gravity-resisted shoulder and elbow exercises under therapist supervision. **(b)** Home-based follow-up: patient using elastic resistance bands to strengthen the left upper limb. **(c)** Home-based follow-up: patient completing dumbbell resistance training for continued shoulder and arm strengthening.

By day 11 post-rehabilitation, the patient achieved full range of motion in the left upper limb with support against gravity. After discharge, he continued with a structured home program. The rehabilitation protocol remained unchanged except for the inclusion of slow-speed resistance training using elastic devices. All sessions were conducted under consistent conditions following a structured protocol (Figure 4), with three sets of 30 repetitions for each of the following exercises.

1st set, slowly move the elbow joint through flexion and extension, then hold the end position for 10 s.

2nd set, slowly move the shoulder joint through forward flexion, extension, horizontal abduction, and horizontal adduction, then hold the end position for 10 s.

3rd set, Perform actively assisted exercises to maintain the range of motion of the left upper extremity.

One month post-intervention, the patient attended a follow-up visit, showing full left upper limb range of motion against gravity and mild resistance, with occasional fatigue. The Gilbert Score and MRC-SS were reassessed (Table 3), and the rehabilitation protocol continued with only a minor adjustment to the motion angle. Two months post-intervention, the patient could lift 6-pound dumbbells. Repeated NCS revealed normal sensory conduction velocities, needle EMG showed normal insertional activity, and motor conduction velocities were also normal, although the motor amplitudes of the left musculocutaneous and suprascapular nerves were slightly reduced compared with the right. Physical examination revealed normal joint mobility, nearly normal gait, intact sensation in the left upper limb, and a MRC grade of 5/5. After six months of rehabilitation, the patient's left upper limb motor impairment had fully resolved, allowing unrestricted physical activities and indicating a successful recovery.

## 3 Discussion

Perioperative PNI following general anesthesia typically arises from multifactorial causes, including local factors such as stretching, compression, ischemia, and transection, and systemic factors like hypotension and inflammation (11). Consequently, a comprehensive neurological examination should be conducted as soon as the patient is sufficiently cooperative. Delayed diagnosis may negatively affect the long-term recovery and outcome of perioperative PNI.

Research shows that in animal models, histological nerve damage correlates with compression intensity and duration (12). Similarly, surgical factors like prolonged operative time and patient positioning during anesthesia elevate perioperative PNI risk. The brachial plexus is highly susceptible, with injuries reported in supine, lateral, Trendelenburg, and prone positions (13). One week before the symptom onset, the patient underwent a 4-hour laparoscopic appendectomy under general anesthesia with a modified Trendelenburg position, a duration exceeding the typical standard (14), which significantly elevates traction and compression forces on the brachial plexus. After ruling out other causes, we diagnosed an iatrogenic BPI due to intraoperative traction.

Determining the specific injured segment after identifying the cause of the injury is crucial for designing a targeted rehabilitation protocol and predicting prognostic outcomes. Iatrogenic BPIs caused by intraoperative patient positioning can be classified as affecting the upper, middle, or lower trunk, with upper and middle trunk injuries typically manifesting as motor deficits in the C5-C6 myotomes, often without sensory deficits. Common clinical signs include weakness in shoulder abduction and elbow flexion (4), consistent with this patient's presentation. NCS and needle EMG are essential postoperative diagnostic tools for peripheral PNI (15), facilitating confirmation of the diagnosis and localization of the site of injury. A thorough physical examination combined with electrophysiological findings supported involvement of the C5–C6 (upper trunk) region. This localization was based on the characteristic clinical pattern of selective weakness in shoulder abduction and elbow flexion with relative preservation of distal motor and sensory function, together with neurogenic changes identified in corresponding muscle groups.

However, MRI showed no significant morphological abnormalities of the brachial plexus, which does not exclude brachial plexus injury and highlights the recognized limitations of conventional MRI in evaluating the plexus, particularly when injuries are subtle and lack overt structural disruption (16). This limitation is partly related to the complex anatomy and oblique course of the brachial plexus, as well as the limited conspicuity of subtle intraneural signal changes on routine sequences (17, 18). Recent studies have demonstrated that advanced MR neurography techniques, including 3D SHINKEI and contrast-enhanced MR neurography, can improve visualization of brachial plexus abnormalities compared with conventional MRI, suggesting that a negative routine MRI should be interpreted with caution rather than as evidence against injury (18, 19). In the present case, MRI was therefore more useful for excluding alternative structural explanations, including cervical radiculopathy, compressive lesions, or gross plexus disruption. Consequently, lesion localization to the C5–C6 (upper trunk) level should be considered a clinically and electrophysiologically supported inference rather than a direct imaging-based confirmation.

According to the Sunderland classification (20), this case was most consistent with a second-degree injury (axonotmesis), affecting primarily motor fibers while relatively sparing sensory fibers. This classification is supported by the severe motor deficits, electrophysiological evidence of denervation in affected muscles, and gradual functional recovery over months. In contrast, neurapraxia involves transient conduction block without denervation and typically resolves more rapidly, which is inconsistent with this patient's clinical and electrophysiological course. Given the potential for axonal regeneration, nerve function may recover over months, indicating a favorable prognosis with appropriate rehabilitation.

This case underscores the importance of identifying the precise etiology of PNI and illustrates the prognostic trajectory of iatrogenic BPI. The novelty of this report relative to existing literature lies in three key aspects. First, it describes a rare iatrogenic C5–C6 BPI in an adolescent patient during laparoscopic surgery—a demographic and surgical setting less commonly reported than adult orthopedic or cardiothoracic cases. Second, the intervention timeline and structure are distinct: whereas rehabilitation is often delayed until spontaneous recovery plateaus, our protocol was initiated within one week of diagnosis. This very early intervention used a structured multimodal approach that progressed smoothly from passive therapist-guided exercises to active progressive resistance training with elastic bands. Third, this case provides practical evidence that early standardized rehabilitation can achieve full functional recovery within 6 months. Importantly, it demonstrates that even when spontaneous axonal regeneration is possible, early multimodal therapy is essential to prevent secondary complications such as muscle atrophy and joint contractures, which frequently impair long-term functional outcomes in BPI.

Structured early multimodal rehabilitation yielded clear functional improvements in the affected upper limb, including gains in muscle strength and active range of motion. Within this regimen, slow-speed resistance training using portable elastic devices offers particular advantages: it closely mimics functional movement, is cost-effective, and supports long-term adherence to home-based programs.

However, the favorable recovery in this case should be interpreted with caution. In addition to the rehabilitation program, the patient also received several pharmacological treatments, including methylcobalamin, adenosine cobalamin, nerve growth factor, ganglioside, and dexamethasone. These agents may have contributed to recovery through neurotrophic, neuroprotective, or anti-inflammatory effects. Because pharmacological therapy and rehabilitation were administered concurrently, their respective contributions cannot be clearly separated. Moreover, some degree of recovery may occur spontaneously in incomplete brachial plexus injuries, particularly in upper trunk injuries or cases with milder injury patterns, further limiting causal inference in a single-case report. Nevertheless, even when spontaneous nerve recovery is expected, early rehabilitation may still be clinically justified to help prevent secondary complications related to reduced limb use (such as muscle atrophy and joint contracture), maintain joint mobility, promote muscle activation, reduce disuse-related weakness, support a safe return to activity, and facilitate monitoring of recovery progression (6, 21). Therefore, the favorable outcome observed here is more appropriately interpreted as the result of a combined therapeutic approach, rather than being attributed solely to rehabilitation. Further studies with larger samples are needed to clarify the relative contributions of rehabilitation and pharmacological treatment and to establish a standardized management strategy for BPIs of varying severities.

## 4 Patient perspective

The patient conveyed profound gratitude to the medical staff of our department for enabling a prompt and definitive diagnosis and treatment. Throughout the rehabilitation training, the patient sustained close communication with the attending physician. Positive feedback on training outcomes, combined with the physician's diligent guidance, fostered high patient compliance, establishing a virtuous cycle during the treatment process.

## Data Availability

The original contributions presented in the study are included in the article/Supplementary Material, further inquiries can be directed to the corresponding authors.
